# Minimal set of nodes to control the dynamics of biological neuronal networks

**DOI:** 10.1186/1471-2202-16-S1-P135

**Published:** 2015-12-18

**Authors:** Simachew Mengiste, Ad Aertsen, Arvind Kumar

**Affiliations:** 1 Faculty of Biology and Bernstein Center Freiburg, University of Freiburg, Freiburg im Breisgau, Germany; 2 Computational Biology, School of Computer Science and Communication, KTH, Stockholm, Sweden

## 

Controlling the dynamics of biological neuronal network (BNNs) is of great importance for both understanding the brain and for developing means to control brain disease related aberrant activity. In the last decade, the relationship among node properties, network structure and network dynamics has become clearer and several neuron and network properties could be used to control the network dynamics. Designing appropriate control inputs would require selective manipulation of the control variable in each node. However, the large size of BNNs and scarcity of resources do not allow control of all individual nodes by external means. Therefore, there is a need to identify a much smaller number of influential nodes that are sufficient to drive the activity dynamics of the entire BNN to a desired state.

Here, we used the concept of structural controllability [1] to identify a small set of influential nodes, *control nodes*, for synthetic and experimental networks. The later includes large-scale connectivity of macaque cortical regions [2], meso-scale connectome of a mouse brain [3] and the neuronal connectivity of *C. elegans *[4]. We investigated the effect of random and systematic edge pruning on the number of these control nodes. Moreover, We showed that there is a power law or exponential dependence between the number of control nodes and the average degree of many networks. Finally, we used a simplified network of the basal ganglia to qualitatively show that few control nodes can indeed drive the network dynamics to a desired state. While every sub-network could be used as a control node or sensor, each node is different in terms of the required stimulation pattern, stimulation energy and time to reach the desired dynamical state.
